# Construct validity and reliability Amharic version of perceived stress scale (PSS-10) among Defense University students

**DOI:** 10.1186/s12888-022-04345-9

**Published:** 2022-11-09

**Authors:** Bitew Sintayehu Tsegaye, Amanuel Kidane Andegiorgish, Abebe Feyissa Amhare, Habtamu Belay Hailu

**Affiliations:** 1 Defence University, College of Health Sciences, Bishoftu, Ethiopia; 2 Salale University, Fitche-Salale, Ethiopia; 3 Department of Epidemiology and Biostatistics, School of Public Health, Xi’an Jiaotong University Health Science Center, Xi’an, China; 4 Department of Epidemiology and Biostatistics, Asmara College of Health Sciences, School of Public Health, Asmara, Eritrea

**Keywords:** Perceived stress scale, Validity, Reliability, University students, Ethiopia

## Abstract

**Background:**

Perceived stress scale (PSS) is the most widely used tool for assessing stressful life events and its management. However, its validity and Reliability in Ethiopian Amharic language is not assessed.

**Objective:**

To translate the perceived stress scale (version PSS-10) and assess its validity among Defense University students in Bishoftu, Ethiopia.

**Method:**

From March to May 2020**,** an anonymous, self-managed questionnaire was used to collect the data on 758 undergraduate students of Defense University in Bishoftu, Ethiopia. Exploratory and Confirmatory factor analyses were employed to assess the factor structure and construct validity of Amharic version of the PSS-10. Composite reliability coefficient and Item total correlation were calculated to assess the internal consistency of Amharic version of the PSS-10.

**Result:**

Exploratory factor analysis resulted in a two-dimensional PSS-10 with Eigenvalues of 3.4 and 1.6, which explained 50.7% of the variance. Confirmatory factor analysis indicates a good model fit of the two correlated factors (Comparative fit index (CFI) = 0.96 with root mean square error of approximation (RMSEA) = 0.04[0.03–0.06] and standardize root mean residual (SRMR) = 0.040). The internal consistency of PSS-10 and the Negative factor were in acceptable range, whereas the Positive factor was marginally acceptable (0.77, 0.78, and 0.68) respectively.

**Conclusion:**

The Amharic translated version of PSS-10 was found to be a valid and reliable instrument to measure the perceived stress level among university students.

## Background

Stress is the interplay of a person with ones environment which is deemed by the individual as menacing or affecting ones potential, resource and wellbeing [1]. To that end, for a stress response to be occurred, there should always be coactions among internal and external factors. As a result, the same stimulus might trigger inconsistent responses among individuals. With regard to individual copying styles, manifestation of people to stress is commonly varied in each circumstance which is determined by past experience, individual characteristics, and prior copying history [2].

Tertiary level education may impose stress on students from many sources of stress such as different environments, lifestyle changes, academic burdens, and interpersonal relationships, all of which can lead to significant psychological dysfunctions [3].

There is a report of the rise in the prevalence of perceived stress in the course of professional study [4]. Previous studies reported a high incidence of perceived stress among higher education students, resulting poor academic performance, mental distress, withdrawal, and other health-related problems [5].

Several instruments had been employed to estimate the stress experienced by university students, Cohen and Williamson (1988) developed the perceived stress scale (PSS-10), which measures the degree to which one perceives aspects the degree to which one perceives aspects of one’s life as uncontrollable, unpredictable, and overloading. The original PSS comprises 14 items (PSS-14). Two shorten versions (PSS-10 and PSS-4) are also available which comprise 10 and 4 items selected from the PSS-14 respectively [6, 7].

The original PSS-10 was considered as a single construct. However, previous studies reported the presence of two dimensions of PSS-10 using principal component analysis [7–10]. In addition, confirmatory factor analysis in other studies indicated the two-factor dimensionalities appear to be a better fit [11, 12].

Several previous researches have examined the factor structure, validity, and reliability of PSS-10 among university students. A study conducted among Mezan-Aman University students in Ethiopia using the original PSS-10 found that the two-factor model is the most suitable to assess perceived stress [13]. Similarly, in another study conducted among Chinese nursing university students to assess the psychometric property of PSS-10, the finding revealed adequate validity and reliability of the two-factor models [14]. In addition, a study among Chinese and Japanese university students also reported a two-factor model PSS-10 and its adequate validity and reliability [15, 16].

The PSS-10 has been translated into several languages including Czech, Arabic, and Vietnamese [15, 17, 18]. As well as across many populations including chronic disease patients, military personal, and university students [19–22].

To the knowledge of the researchers, the perceived stress scale is not translated and validated in Amharic; a national language of more than 110 million populations. Therefore, the aim of this study is to translate and validate the Amharic version PSS-10 among military university students in Ethiopia.

## Methods

An institutional based cross-sectional study was conducted using a self-administered questionnaire. The students were asked to complete a set of questions consisting of two parts: demographic information and the PSS-10. The study was conducted at Defense University. Undergraduate students have been recruited from three colleges of Defense University: Health Science, Resource Management, and Engineering Colleges.

A total of 758 students from the three colleges in accordance with their proportional size were participated in this study and within each college, all respective departments have been included. As to the sampling technique, the study units were selected from each department and year of study using simple random sampling proportional to size.

### Instruments

This particular PSS-10 has a 5-point Likert scale ranging from 0 (never) to 4 (very often), indicating how often respondents have felt stressed in a certain way in their life within the past month. Six out of 10 items of the PSS-10 were worded as the negative questions (1, 2, 3, 6, 9, 10) and the remaining four items as positive (4, 5, 7, 8), representing “perceived distress” and “perceived coping”, respectively [6]. Scores can range from 0 to 40. Participants with higher scores are regarded to have higher perceived stress levels [7]. The PSS-10 was translated from the original English version into Amharic by two English-Amharic bilingual psychologists who did not know the wording of the original English version of the PSS. The two English versions were then compared item-by-item and minor discrepancies were addressed and corrected in the Amharic version by a consensus of these translators. The Amharic version of PSS-10 was piloted among 30 private medical college undergraduate students. Further corrections to the translation were completed based on the results of this pilot study. Ethical clearance was obtained from the Research Ethics Committee of Defense University health science college.

### Statistical analysis

Data management and analysis were done using STATA software version 14.0. Continuous variables were presented as mean and standard deviation whereas categorical variables were presented as frequency and percentage.

The internal consistency reliability of the Amharic version PSS-10 and its subscale was examined by composite reliability coefficient which is not required the tau equivalent model assumption [23], as such a value of greater than or equal to 0.7 indicated sufficient reliability. Furthermore, Item Total Correlation has been calculated to confirm internal consistency.

To analyze the construct structure of the Amharic version of PSS-10, the sample was randomly split into two halves. An Exploratory Factor Analysis (EFA) and Confirmatory Factor Analysis (CFA) were conducted with the first and second halves, respectively. The EFA was performed with principal component extraction method. The Kaiser-Meyer-Olkin measure of sampling adequacy was applied to assess sample adequacy prior to the EFA. Eigenvalue was used to decide the number of factors to retain and eigenvalue greater than one were retained. Two-factor structure of PSS-10 was evaluated through CFA. The covariance matrix was tested by the maximum-likelihood estimation method to determine how well the model fitted the sample data. Comparative Fit Index (CFI) values > 0.95, Root Mean Square Error of Approximation (RMSEA) with 90% confidence intervals and value < 0.06, a non-significant chi-square indicate that the model is a good fit [24, 25]. *P* < 0.05 was considered statistically significant.

## Results

### Demographic characteristics of respondents

Among 758 undergraduate students who participated in the study, 423(55.8%) were social science and engineering students and 335(44.2%) were health science students. Mean (±SD) age of the study participants was 26.3 ± 5.8 years, with majority 663 (87.4%) were males.

Regarding their year of study; 192 (25.3%), 177(23.4%), 146(19.3%), 105(13.9%) and 138(18.2%) of the participants were first year, second year, third year, fourth year and fifth year students, respectively. Overall mean perceived stress score of the whole study participants was 19.4 ± 4.2[CI: 19.2–19.7].

### Internal consistency of PSS-10

Composite reliability coefficients for the factors were 0.77, 0.78, and 0.68 for the total scale, negative factor, and positive factor, respectively. Item-rest correlation (The correlation of an item with a total of the remaining items) was also computed and in an acceptable range (Table 1).

**Table 1:** Descriptive statistics and Item-rest correlation of PSS-10

PSS-10 Items	Mean ± SD	Item-rest correlation (Total PSS)	Item-rest correlation	
			Negative Factor	Positive Factor
PSS-1	1.6 ± 1.06	0.40	0.49	
PSS-2	1.3 ± 1.06	0.52	0.57	
PSS-3	1.7 ± 1.06	0.52	0.58	
PSS-4	2.4 ± 0.98	0.35		0.43
PSS-5	2.1 ± 0.96	0.48		0.46
PSS-6	1.4 ± 1.01	0.50	0.40	
PSS-7	2.6 ± 0.95	0.27		0.39
PSS-8	2.6 ± 1.04	0.41		0.49
PSS-9	1.7 ± 1.01	0.52	0.55	
PSS-10	1.7 ± 1.07	0.46	0.51	
Total	15.7 ± 5.9			

### Factor structure and construct validity of PSS-10 item

Bartlett’s test of sphericity was 866.666 (*p* < 0.001) and Kaiser-Meyer-Olkin measure of the sampling adequacy was 0.82, supporting the use of these data in factor analysis for further investigation.

The explanatory factor analysis with Varimax rotation of all 10 items in the PSS-10 scale yielded two factors with an initial Eigenvalue of > 1.0, the eigenvalue of negative and positive factors were 3.4 and 1.6 respectively. The two-factor solution was found to be 50.7% of the variance; for which the first factor was accounted for 29% of the variance and the second factor for 21.6% of it. As to Factor Loading, items were found to be ranged from 0.58–0.74. The items and their loadings on each factor are presented in Table 2.

**Table 2:** Exploratory factor analysis of Amharic version of PSS-10 in Defense university students

Items description	Negative Factor	Positive Factor
In the last month, how often have you been upset because of something that happened unexpectedly?	0.70	
In the last month, how often have you felt that you were unable to control the important things in your life?	0.72	
In the last month, how often have you felt nervous and stressed?	0.74	
In the last month, how often have you found that you could not cope with all the things that you had to do?	0.58	
In the last month, how often have you been angered because of things that happened that were outside of your control?	0.68	
How often have you felt difficulties were piling up so high that you could not overcome them in the last month?	0.66	
How often have you felt confident about your ability to handle your problems in the last month?		0.71
How often have you felt that things were going your way in the last month?		0.65
How often have you been able to control irritations in the last month?		0.70
How often have you felt that you were on top of things in the last month?		0.73
Variance percent (%)	29.0%	21.6%
Total variance (%)	50.7%	
Bartlett test of sphericity 866.666 (*P*-value < 0.001)		
Kaiser-Meyer-Olkin measure of the sampling adequacy (KMO) 0.82		
Eigenvalue	3.4	1.6

**Fig. 1:**
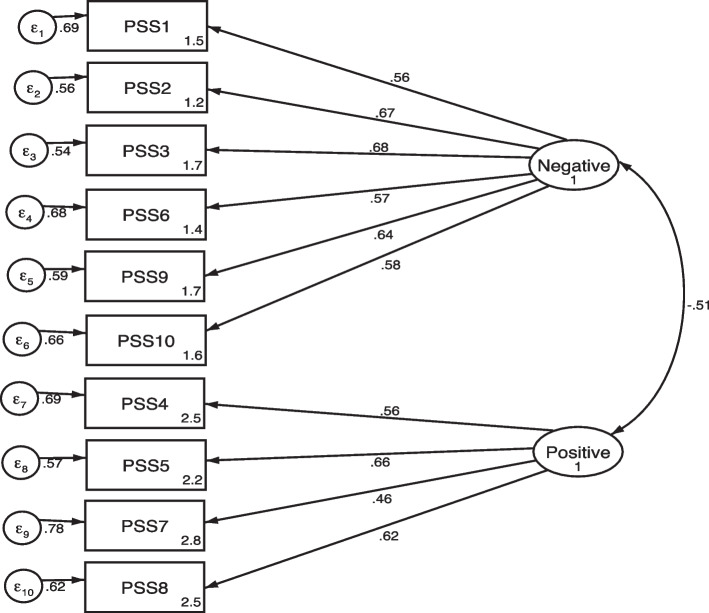
Standardize factor loading of 2-factor PSS-10

All factor loadings for two-factor model were found to be significant and in the expected direction, ranging from 0.40 to 0.67 (Table 3) and (Fig.1).

**Table 3:** Confirmatory factor analysis of the two-factor model of PSS-10

Item description	Two-factor model	
	Negative factor	Positive factor
How often have you been upset because of something that happened unexpectedly in the last month?	0.57	
In the last month, how often have you felt that you were unable to control the important things in your life?	0.67	
In the last month, how often have you felt nervous and stressed?	0.66	
In the last month, how often have you found that you could not cope with all the things that you had to do?	0.56	
In the last month, how often have you been angered because of things that happened that were outside of your control?	0.61	
How often have you felt difficulties were piling up so high that you could not overcome them in the last month?	0.59	
How often have you felt confident about your ability to handle your problems in the last month?		0.50
How often have you felt that things were going your way in the last month?		0.68
How often have you been able to control irritations in the last month?		0.40
How often have you felt that you were on top of things in the last month?		0.63
Factor Correlation	0.57	
Composite reliability	0.78	0.68

The CFA was used to evaluate the goodness of fit of the two-factor models of PSS-10. The fit indices showed that the two-factor model was a good fit to the data (X^2^/df = 1.9; CFI = 0.96; RMSEA = 0.04[0.03–0.06] and SRMR = 0.040) (Table 4).

**Table 4:** Results of CFA of model testing of Amharic version PSS-10

MODEL	*X*^*2*^	DF	*X*^*2*^*/df*	P-value	CFI	RMSEA	SRMR
2-Factor model	64.40	34	1.9	0.0013	0.96	0.04[0.03–0.06]	0.040

*CFI* Comparative fit index, *RMSEA* Root mean square error of approximation, *SRMR* Standardize root mean residual

## Discussion

The current study findings indicated that the Amharic version PSS-10 is a reliable and valid instrument for the assessment of perceived stress among university students in Ethiopia.

Internal consistency for the total PSS-10 and the negative subscale were found to be acceptable (> 0.7). On the other hand, for the positive subscale, the study showed that it was marginally satisfactory (0.68). In addition, the Item-rest correlations were also in an acceptable range indicating the direct contribution of individual items towards the total score on the PSS-10. A lower internal consistency coefficient of the positive factor has also been reported in previous studies of PSS-10 conducted in other languages [9, 13, 19].

The findings further revealed that the correlation coefficient between the two factors was significant (*r* = 0.57); indicative of good internal homogeneity. Since both factors reflect perceived stress, it has been suggested that any distinction between these factors is irrelevant and reflects the sentence structure of the scale [15]. The findings of this particular study were found to be consistent with previous findings [15, 18]; hence it can be concluded that using the total PSS-10 scale is preferable rather than computing the two factors separately as it was recommended by Cohen’s (the original developer of the scale) to use all 10 items in order to measure perceived stress [26].

Regarding the Amharic version of the PSS-10 factor structure, the EFA analysis showed that the Amharic version of PSS-10 corroborated the two-factor structure and is in line with the original study and previous studies of PSS-10 carried out in other languages. As to the two factors, the factor with 6 items which were negatively worded the factor loading was found to be in the range of 0.57–0.74 while the positively worded factor having 4 items with the factor loading of 0.65–0.73. All items had loadings above 0.5 on one of the two factors which have been identified in this study indicated that all of them contributed significantly to measuring the perceived stress concept among university students. Several studies have been conducted to examine the factor structure of PSS-10: the findings among military sample in Korea, a study done among Turkish university students, the Arabic version of PSS-10 among pregnant women, a similar study among Chinese policewomen, and the study conducted among early child teachers in South Korea, all of which demonstrated a two-factor structure [14, 17, 19, 20, 27]. Moreover, a study conducted among Meza-Aman University students in Ethiopia using the original English version further confirmed that the two-factor structure [13].

In order to examine the validity of Amharic version PSS-10, CFA analysis was conducted. Accordingly, the result of the CFA analysis revealed that, the two-factor model demonstrated a good fit. Correspondingly, the findings of this study were found to be in line with the original version and previous findings using the PSS-10 versions as well [12–14, 17, 26].

### Limitations of the study

Several limitations of this study are worth to be noted. The scale was tested for psychometric properties in university students and therefore the generalization of the current study may only be applicable for similar population groups. Second, the current study is based on self-reported measures, hence reporting bias might be occurred. In addition, the data was collected using cross-sectional design and therefore predictive validity and test-retest reliability of PSS-10 could not have been computed.

## Conclusion

The findings of this study revealed that the Amharic version PSS-10 could be a valid and reliable instrument with adequate psychometric properties. Therefore, the Amharic version PSS-10 can be a very useful instrument to measure psychological stress among Amharic speaking population and also used to measure perceived stress in future researches and practices among university students. As the PSS-10 has now been translated to more than 20 languages, the use of Amharic version PSS-10 will provide additional opportunities for cross-cultural comparison. However, further studies are recommended to further endorse the validity and reliability of PSS-10.

## Data Availability

Pertinent data are available in this manuscript. Additional data can be requested from the corresponding author upon reasonable request.

## Abbreviations

PSS: Perceived Stress Scale
EFA: Exploratory Factor Analysis
CFA: Confirmatory Factor Analysis
RMSEA: Root mean square error of approximation
SRMR: Standardize root mean residual
CFI: Comparative fit index
